# Population genomics of the endangered giant Galápagos tortoise

**DOI:** 10.1186/gb-2013-14-12-r136

**Published:** 2013-12-16

**Authors:** Etienne Loire, Ylenia Chiari, Aurélien Bernard, Vincent Cahais, Jonathan Romiguier, Benoît Nabholz, Joao Miguel Lourenço, Nicolas Galtier

**Affiliations:** 1 Université Montpellier 2, CNRS UMR 5554, Institut des Sciences de l’Evolution de Montpellier, Place E. Bataillon, 34095 Montpellier, France; 2 CIBIO, Centro de Investigação em Biodiversidade e Recursos Genéticos, Universidade do Porto, Campus Agrário de Vairão, 4485-661 Vairão, Portugal

## Abstract

**Background:**

The giant Galápagos tortoise, *Chelonoidis nigra*, is a large-sized terrestrial chelonian of high patrimonial interest. The species recently colonized a small continental archipelago, the Galápagos Islands, where it has been facing novel environmental conditions and limited resource availability. To explore the genomic consequences of this ecological shift, we analyze the transcriptomic variability of five individuals of *C. nigra*, and compare it to similar data obtained from several continental species of turtles.

**Results:**

Having clarified the timing of divergence in the *Chelonoidis genus*, we report in *C. nigra* a very low level of genetic polymorphism, signatures of a weakened efficacy of purifying selection, and an elevated mutation load in coding and regulatory sequences. These results are consistent with the hypothesis of an extremely low long-term effective population size in this insular species. Functional evolutionary analyses reveal a reduced diversity of immunity genes in *C. nigra*, in line with the hypothesis of attenuated pathogen diversity in islands, and an increased selective pressure on genes involved in response to stress, potentially related to the climatic instability of its environment and its elongated lifespan. Finally, we detect no population structure or homozygosity excess in our five-individual sample.

**Conclusions:**

These results enlighten the molecular evolution of an endangered taxon in a stressful environment and point to island endemic species as a promising model for the study of the deleterious effects on genome evolution of a reduced long-term population size.

## Background

Evolution on islands is a fascinating topic. A number of plant and animal species are known to be endemic from small islands or archipelagos, having evolved in isolation from their continental relatives during long periods of time. Such systems are typically seen as natural laboratories for the study of adaptation [1]. Invading an island means entering a new biotic environment, that is, a new community of competitors, predators, preys and parasites, and a reduced total amount of available food. This sudden ecological challenge must be faced by a supposedly small number of migrants, in a context of reduced or null gene flow from the mainland. The successful colonization of an island by a new species is therefore likely to be driven by rapid adaptive evolution. Consistently, evolution on islands is often associated with rapid morphological changes [2], the observation of which has been of major importance in Darwin’s thoughts and conceptions. In the genomic era, the search for the molecular targets of such adaptive processes appears as a promising quest.

A second reason why island endemic species are of specific interest to evolutionary biologists is their supposedly reduced population size. The effective population size (*N*_e_) is a central parameter of the population genetic theory, which determines the strength of genetic drift, the random fluctuation of allele frequencies generation after generation. The theory makes a number of important predictions regarding the influence of *N*_e_ on patterns of molecular diversity. First, small populations are expected to be genetically less diverse than large populations because of the reduced sojourn time of neutral mutations in the former. The existing data seem in broad agreement with this prediction at a wide phylogenetic scale [3,4]. In studies of more restricted taxonomic groups, a relationship between population size predictors and genetic diversity has been reported in fish [5], but not in mammals [6] or birds [7], despite abundant genetic data in the latter two taxa.

Importantly, genetic drift is also expected to decrease the efficiency of natural selection, as it pushes the frequency of an allele up and down irrespective of its contribution to fitness. Consequently, natural selection in favor of slightly advantageous mutations and in disfavor of slightly deleterious mutations is supposed to be less efficient in small than in large populations [8]. It was convincingly argued that the *N*_e_ effect is the major explanation for the difference in genome architecture between prokaryotes and large organisms [9]. Besides this contrast, it would appear important to determine whether the *N*_e_ effect on selection efficiency is detectable at a more recent phylogenetic scale, that is, between closely-related species. In particular, whether species affected by a recent drop in population size are ‘genetically endangered’ (that is, suffer from an increased load of deleterious mutations) is still debated [6,10].

To date, empirical evidence regarding the influence of *N*_e_ on the efficiency of natural selection is not so abundant. The most convincing contribution was the report in mammals of a positive correlation between body mass and the ratio of non-synonymous to synonymous substitutions (*d*_N_/*d*_S_) [11]. An increased *d*_N_/*d*_S_ ratio is expected when the efficiency of purifying selection against deleterious non-synonymous changes is weakened. The mammalian pattern was therefore interpreted as a *N*_e_ effect, plausibly assuming that populations of large animals tend to be smaller than populations of small animals, on average. Still in mammals, the evolutionary rate of non-coding sequences upstream and downstream of genes was reported to be faster in primates than in rodents, which was interpreted in terms of a reduced *N*_e_ in primates [12].

One problem for testing the population size effect on patterns of molecular variation is that we typically have no direct measurement of *N*_e_, which in the above-cited studies was indirectly approached through various surrogates (for example, body mass, conservation status, marine *vs*. terrestrial habitat). The long-term average *N*_e_, which is the relevant parameter in molecular evolution, may be badly predicted by current species abundance [6,7]. Islands provide a good opportunity to cope with these problems: island endemic species are likely to have evolved in small populations during long periods of time, owing to the overall limitation in space and resource availability. Consistent with this prediction, an increased *d*_N_/*d*_S_ ratio for mitochondrial genes was reported in island endemic species, as compared to their mainland relatives [13,14], which was again interpreted as reflecting a weakened efficiency of purifying selection due to a smaller long-term *N*_e_.

The signature of a reduced *N*_e_ in the islands, although significant, was only observed in approximately 60% of the island/mainland couples [14], perhaps because of a lack of power - just one or two genes were analyzed for each species pair (and see reference [15]). It should also be noted that the *d*_N_/*d*_S_ ratio, used in most of the above-mentioned studies, is not only determined by the strength of purifying selection, but also by the rate of adaptive amino-acid substitutions, which in some species can be far from negligible [16]. If adaptation on islands was a prominent process at the molecular level too, then the *d*_N_/*d*_S_ ratio might be inflated independently of demographic effects. In this case, the ratio of non-synonymous over synonymous polymorphism (π_N_/π_S_), not divergence, would be a more appropriate marker of a reduced *N*_e_, since it is much less affected by adaptive evolution than the *d*_N_/*d*_S_ ratio [17]. The availability of within-species variation data is therefore of primary importance to disentangle the adaptive *vs*. non-adaptive forces driving molecular evolution on islands.

Here we present a population genomic study of the giant Galápagos tortoise, *Chelonoidis nigra*, an endangered species endemic from the Galápagos archipelago. Mitochondrial DNA (mtDNA) analyses suggested that this insular species has been isolated from the South American continent during millions of years [18,19]. *C. nigra* is together with the Aldabra tortoise the largest known living species of terrestrial turtles, much larger than its mainland congenerics, and can live well above 100 years. Its new environment, the Galápagos archipelago, is affected by strong climatic fluctuations in space and time, with some islands being generally quite arid, and all of them experiencing long periods of drought associated to ‘El Niño’ southern oscillations [20]. *C. nigra* is therefore a perfect model for the study of adaptation following island colonization. On the other hand, its large size combined to its endemic status is suggestive of a small long-term *N*_e_ for this species, which might have favored non-adaptive evolution through reinforced genetic drift. To test these hypotheses and quantify the relative influence of positive *vs*. purifying selection, the transcriptomic diversity of five individuals was investigated and compared to similar data gathered in several continental species of turtle.

## Results

### Datasets

Five *C. nigra* individuals from three distinct subspecies were analyzed (Table 1, Additional file 1: Figure S1). Besides *C. nigra*, samples from the congeneric red-footed tortoise *C. carbonaria* and from the Spanish pond turtle *Mauremys leprosa* (one individual each) were also collected. The dataset was completed by transcriptome data from the previously published European pond turtle *Emys orbicularis* (10 individuals) and pond slider *Trachemys scripta* (three individuals, Table 2).

**Table 1:** Specimens sampled in this study

**Species**	**id**^**a**^	**id2**^**b**^	**id3**^**c**^	**Sampled in**	**mtDNA**^**d**^	**Reads (*n*)**	**Mbp**
*C. nigra*	GA05A		SRS509366	Rotterdam Zoo (Netherlands)	clade *c,* PBL *(becki)*	8,995,838	793
*C. nigra*	GA05G	zuz10 or zuz20	SRS509367	A Cupulatta Corsica (France)	clade *c,* PBL *(becki)*	10,770,970	987
*C. nigra*	GA05H	zuz10 or zuz20	SRS509368	A Cupulatta Corsica (France)	clade *c,* PBL *(becki)*	10,247,396	940
*C. nigra*	GA05E	zuz30	SRS509369	Zurich Zoo (Switzerland)	clade *d,* VA *(vandenburghi)*	12,862,334	1,143
*C. nigra*	GA05F	zuz01	SRS509370	Zurich Zoo (Switzerland)	clade *d,* CRU *(porteri)*	4,927,381	440
*C. carbonaria*	GA05D		SRS509371	Montpellier Zoo (France)		9,218,341	831
*M. leprosa*	GA03B		SRS509372	Banyuls (France)		9,903,466	885

^a^This study.

^b^According to Russello et al. [21].

^c^NCBI SRA accession id.

^d^Lineage according to Caccone et al. [18] and Russello et al. [21].

**Table 2:** Turtle transcriptomic sequence datasets analyzed in this study

**Species**	**Family**	**Ind**	**Tissue**	**454**	**Illu.**	**Contigs**^**a**^	**N50**	**ORF**^**b**^	**Ref.**^**c**^
*C. nigra*	Testudinidae	5	Blood	yes	yes	80,693	685	10,929	(1), (2)
*C. carbonaria*	Testudinidae	1	Blood	no	yes	11,324	536	1,236	(1)
*M. leprosa*	Geoemydidae	1	Blood	no	yes	10,199	576	1,439	(1)
*E. orbicularis*	Emydidae	10	Blood	yes	yes	68,096	720	10,503	(3)
*T. scripta*	Emydidae	3	Blood, brain	yes	yes	27,235	667	3,383	(3), (4)
*C. caretta*	Cheloniidae	1	Blood	yes	no	31,135	509	5,142	(2)
*P. hilarii*	Chelidae	1	Blood	yes	no	45,060	563	8,066	(2)

^a^Number of contigs of length >200 bp.

^b^Number of ORF of length >100 codons.

^c^(1): this study; (2) Chiari et al. [22]; (3) Gayral et al. [23]; (4) Tzika et al. [24].

### Turtle phylogenomics

**Figure 1:**
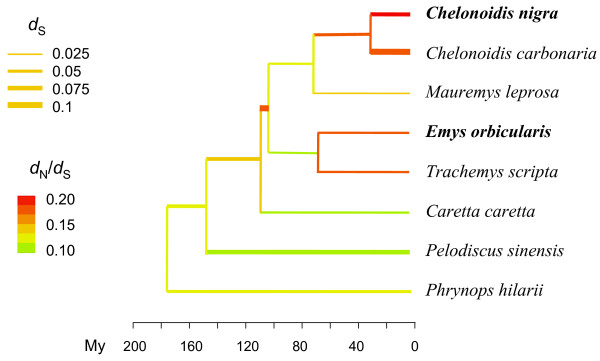
**Maximum-likelihood coding sequence analysis of 248 genes in eight turtle species.** Branch lengths are proportional to time. Branch thickness: number of synonymous changes per million years per 100 synonymous sites. Colors: branch-specific *d*_N_/*d*_S_ ratio.

We first analyzed a dataset of 248 orthologous genes in eight turtle species. The tree topology we obtained (Figure 1) was consistent with published Testudines phylogenies [25,26]. Branch lengths in Figure 1 are proportional to time, whereas branch thickness reflects the estimated per million years rate of synonymous substitution, obtained by dividing synonymous branch lengths by absolute divergence time. The three tropical lineages (*Phrynops*, *Pelodiscus*, and *Chelonoidis*) showed a higher synonymous substitution rate than the marine (*Caretta caretta*) and temperate ones (*Emys*, *Trachemys*, *Mauremys*), consistent with a recent report in turtles of a negative correlation between synonymous substitution rate and latitude in turtles [27].

Colors in Figure 1 reflect lineage-specific *d*_N_/*d*_S_ ratio. The ratio was highly heterogeneous across lineages, as revealed by the significantly better fit of a model assuming one specific *d*_N_/*d*_S_ ratio per branch, compared to a one-ratio model (2*delta log likelihood = 1,472, 15 degrees of freedom, p-val <10^-100^). Interestingly, the *C. nigra* branch was the one showing the highest *d*_N_/*d*_S_ ratio. This appears consistent with the hypothesis of faster non-synonymous evolutionary rate in the islands [14]. However, according to a phylogenetic analysis of five genes in 32 Testudinoidea species [28], the divergence between the *C. nigra* and *C. carbonaria* lineages dates back to approximately 30 million years ago, whereas the colonization of the Galápagos archipelago by *C. nigra* must be younger than 3 to 4 million years, that is, the age of the oldest Galápagos Islands (see Discussion). Whether insularity explains the high *d*_N_/*d*_S_ ratio observed in the *C. nigra* branch is therefore unclear. Within-species data are clearly more appropriate to specifically address this issue.

### *C. nigra* coding sequence polymorphism

**Figure 2:**
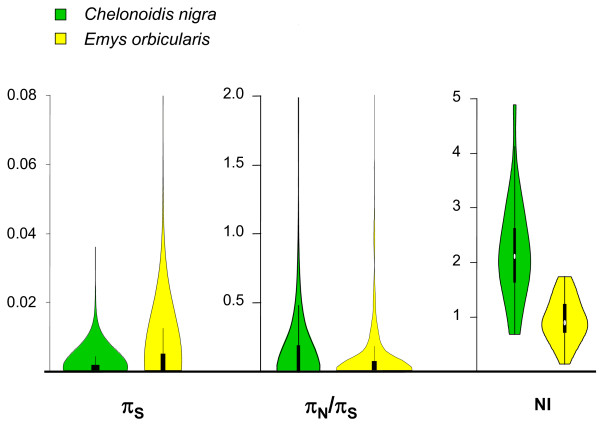
**Distribution of population genomic statistics across predicted coding sequences in*C. nigra*and*E. orbicularis*.** π_N_/π_S_ distribution: coding sequences for which π_S_ = 0 were omitted. NI distribution: coding sequences for which *d*_S_ = 0 were omitted.

In *C. nigra*, individual genotypes and single nucleotide polymorphisms (SNPs) were called using a probabilistic approach paying specific attention to the removal of spurious SNPs due to hidden paralogy. Various conditions were tried regarding the stringency of paralog filtering and the minimal number of genotyped individuals required to validate a SNP (Table 3, see Methods). Only contigs for which an ORF longer than 200 base pairs (bp) was predicted and an ortholog was detected in *C. carbonaria* were included in this (and subsequent) analyses. Between 814 and 1,059 predicted ORFs were analyzed, depending on the settings. They yielded 769 to 1,933 coding SNPs, of which >99.5% were biallelic. Results were reasonably consistent across conditions. Increasing the minimal required number of individuals resulted in a moderate increase in estimated π_S_, and a moderate decrease in π_N_/π_S_ ratio. Increasing the paralog filtering stringency resulted in a decreased π_S_, as expected, but hardly affected the estimated π_N_/π_S_. The sampling variance of these estimates was acceptably low: the width of bootstrap confidence intervals was 10% to 15% of the π_S_ average, and 15% to 20% of the π_N_/π_S_ one. The results corresponding to condition A3 are shown in Figure 2, and compared with a similar analysis conducted in the continental European pond turtle *E. orbicularis* (Table 3, last line).

**Table 3:** Robustness of non-synonymous and synonymous diversity estimates in the giant Galápagos tortoise

**Condition**	**Ind. (*n*)**	**Paralogue filter**	**Genes (*n*)**	**SNPs (*n*)**	**π**_**S**_**(%)**	**π**_**N**_**/π**_**S**_	**F**_**IT**_	**α**_**0.2**_
A1	≥3	Stringent	1,053	1,933	0.18 ±0.03	0.35 ±0.06	-0.14	-0.30
A2	≥4	Stringent	968	1,538	0.18 ±0.03	0.34 ±0.06	-0.12	-0.34
A3	5	Stringent	814	1,041	0.19 ±0.04	0.31 ±0.06	-0.10	-0.15
B1	≥3	Very stringent	1,059	1,423	0.13 ±0.02	0.33 ±0.05	-0.11	-0.05
B2	≥4	Very stringent	974	1,131	0.14 ±0.02	0.31 ±0.05	-0.10	-0.09
B3	5	Very stringent	820	769	0.14 ±0.02	0.28 ±0.05	-0.08	0.18
*Emys*	≥5	Stringent	953	1,845	0.42 ±0.04	0.09 ±0.02	0.21	0.57

The average estimated synonymous diversity (π_S_) in *C. nigra* was 0.0013 to 0.0019, that is, a very low value, similar to the human one [6]. No positive FIT was detected, meaning that the two alleles of a given individual were not more similar, on average, than two alleles sampled in two distinct individuals. Likewise, no significant FST was detected between the two mitochondrial clades sampled in this study, that is, clade *c* (three individuals) and clade *d* (two individuals, Table 1). The estimated average π_N_/π_S_ ratio in *C. nigra* was around 0.3. This is higher than the highest π_N_/π_S_ ratio reported so far (approximately 0.2 in human). The average *C. nigra*/*C. carbonaria d*_N_/*d*_S_ ratio in this analysis was 0.13 to 0.15. This value is similar to the human-specific and chimpanzee-specific dN/d_S_ ratios [6], but still substantially lower than the *C. nigra* π_N_/π_S_ ratio. Consequently, the neutrality index was above unity (Figure 2), even after removal of low-frequency variants, and the proportion of adaptive amino-acid substitution estimated by the McDonald-Kreitman method, α and α_0.2_, was below zero in *C. nigra*. When a method accounting for the whole distribution of allele frequencies was used [29], an estimate of 0.13 was obtained, with a confidence interval including zero. The other turtle species for which we have within-species variation data, *E. orbicularis*, did not show such an extreme population genomic pattern: π_S_ and α were higher, and π_N_/π_S_ and *d*_N_/*d*_S_ lower, in *E. orbicularis* than in *C. nigra* (Figure 2). These results are indicative of an extremely small long-term *N*_e_ in the Galápagos tortoise, and suggest that the elevated rate of non-synonymous substitutions observed in *C. nigra* is in the first place a consequence of increased genetic drift, not accelerated adaptive evolution.

### Flanking sequences

**Figure 3:**
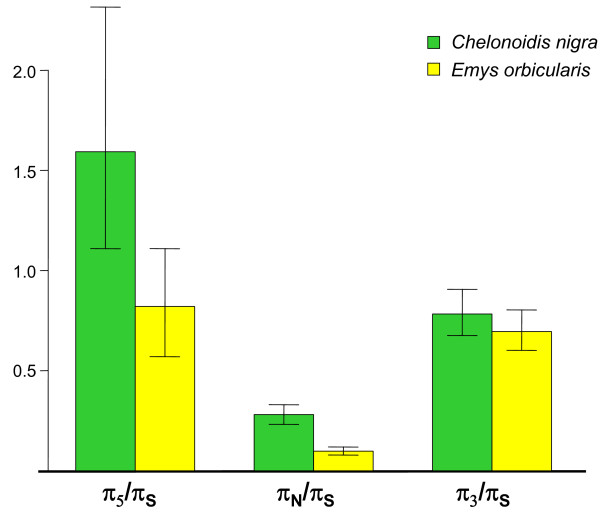
**Flanking *vs*. coding region diversity in *C. nigra *and *E. orbicularis*.** π_S_: average nucleotide diversity at synonymous sites; π_5_: average nucleotide diversity at 5′-UTR sites; π_3_: average nucleotide diversity at 3′-UTR sites. Very similar results were obtained when we only analyzed predicted cDNAs for which both coding sequence and 3′ UTR sequence were available, and when we restricted the analysis to the 500 first bases of the 3′ UTR regions.

To further investigate this hypothesis, we examined the evolution of potential targets of weak selection, namely coding region-flanking sequences. In *C. nigra*, we extracted 158 5′ UTR and 598 3′ UTR sequences of length above 50 bp, and measured levels of genetic diversity based on sites genotyped in at least four individuals (out of five). In *E. orbicularis*, we similarly analyzed 89 5′ UTR and 457 3′ UTR sequences, requiring that at least eight individuals (out of 10) were genotyped. Figure 3 summarizes the relative levels of within-species diversity at 5′ UTR, 3′ UTR, synonymous and non-synonymous sites in the two species. In *E. orbicularis*, the 5′ and especially the 3′ UTR sequences showed a significantly lower amount of diversity than synonymous sites, suggesting that these sequences are under selective pressure. The selective constraint, although detectable, was much less pronounced than for non-synonymous sites, as reflected by the higher π_UTR_/π_S_ than π_N_/π_S_ ratio, in line with similar observations made in mammals [12] and birds [30]. The strength of purifying selection on UTR sequences appeared weaker in *C. nigra*. No significant constraint was detected in 5′ flanking sites, and the estimated level of constraint on 3′ flanking sites was reduced, compared to *E. orbicularis*. This is consistent with the π_N_/π_S_ pattern across species, and with the hypothesis of a reduced *N*_e_ in the Galápagos tortoise.

In neither of the two species did we detect a progressive decrease in the amount of selective constraint with increasing distance to the coding sequence, in contrast with the published mammal and bird analyses [12,30]. We suggest that the difference may result from the fact that we are here analyzing cDNA sequences, whereas the two above-cited studies analyzed genomic sequences. Consequently, our flanking regions only include UTR, whereas theirs included both UTR and untranscribed regions, the proportion of which presumably increases as ones moves away from the coding sequence. For this reason, π_flanking_/π_S_ estimates should not be directly compared between turtles, mammals, and birds across the three studies.

### Gene expression analysis

Gene-specific expression profiles were estimated in the five turtle species for which Illumina data were available. Correlation coefficients of log-transformed, normalized expression levels across genes were calculated for each pair of species. The Testudinidae (*C. nigra*/*C. carbonaria*) and Emydidae (*E.orbicularis*/*T. scripta*) pairs showed the highest level of correlation of gene expression (r2 = 0.78 and r2 = 0.73, respectively). A neighbor-joining analysis of the pairwise correlation matrix produced the ((E. orbicularis, T. scripta), M. leprosa, (C. carbonaria, *C. nigra*)) topology, in agreement with published turtle phylogenies. Branch lengths revealed no specific pattern in the *C. nigra* lineage, which did not show an accelerated evolutionary rate of gene expression pattern. Rather, the *M. leprosa* lineage was the fastest evolving in this analysis (Additional file 2).

### Gene ontology analysis

**Figure 4:**
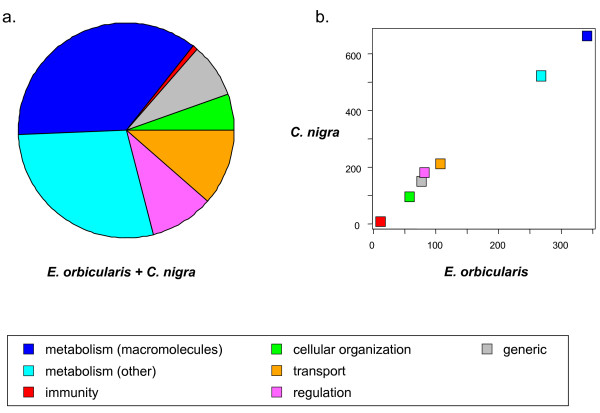
**Functional annotation of coding sequences analyzed in *C. nigra *and *E. orbicularis*. (a)** Pie chart representing the total number of coding sequences from the two species in each category; **(b)** correlation of category-specific abundance between the two species. Only coding sequences for which we obtained both a GO-slim term annotation and a π_N_/π_S_ estimate were included. The generic categories used in this figure were defined by grouping terms from the ‘biological process’ GO-slim ontology as indicated in Additional file 3: Table S1, second colon.

Functional annotation of the *C. nigra* and *E. orbicularis* predicted cDNAs was achieved by sequence similarity using the generic GO-slim ‘Biological process’ gene ontology. Roughly two-thirds of the 2,774 annotated coding sequences (ORFs) were associated to metabolic genes, the other ones being associated to transport, regulation, cellular growth and organization, or immunity (Figure 4, Additional file 3: Table S1). For each GO-slim term, the average log-transformed π_N_/π_S_ ratio was calculated and compared between the two species, in search for terms showing a specific increase or decrease in selective pressure in *C. nigra*. This was achieved through a z-score analysis accounting for sampling variance and global genomic trends (see Material and methods).

**Figure 5:**
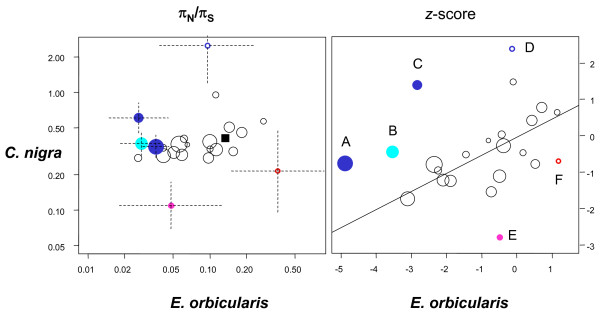
**GO term-specific π**_**N**_**/π**_**S**_**analysis in*C. nigra*and*E. orbicularis*.** Each circle is for a GO-slim term. Circle surface is proportional to the number of associated coding sequences. Left panel: term-average π_N_/π_S_ ratio in log scale; black square: genomic averages. Right panel: term-specific *z*-score. Colored circles: *z*-score significant at the 10% (open) or 5% (close) level. Blue: macromolecule metabolism; cyan: metabolism (other); purple: response to stress; red: immunity. Dotted lines: standard deviation. Letters refer to Additional file 3: Table S1, first colon.

Figure 5 plots the term-specific average π_N_/π_S_ ratio (left panel), and its normalized version (right panel, z-score), in *C. nigra versus E. orbicularis*. A majority of terms show a π_N_/π_S_ ratio lower than the genomic average, and therefore a negative *z*-score. This reflects the fact that coding sequences for which a functional annotation was obtained are more conserved, on average, than the non-annotated ones. Colored circles show the terms for which we detected a significant contrast in π_N_/π_S_ ratio between the two species, as reflected by the Δ_z_ statistics (see Material and methods). Four terms showed a significantly positive Δ_z_, that is, a higher π_N_/π_S_ ratio in *C. nigra* than in *E. orbicularis* relative to genomic averages: ‘RNA metabolic process’, ‘translation’, ‘catabolic process’, ‘nucleotide metabolic process’. Two terms showed the opposite trend, that is, a lower π_N_/π_S_ ratio in *C. nigra*: ‘immune system process’ and ‘response to stress’ (Additional file 3: Table S1).

## Discussion

Genome-wide studies have been so far largely restricted to model organisms, that is, domestic or laboratory species. Here, thanks to NGS technologies, we were able to characterize for the first time the transcriptomic variability and population genomics of an endangered, emblematic species, the giant Galápagos tortoise, in absence of prior genomic knowledge.

One specificity of our sample is the lack of geographic information: we do not exactly know from which locality and each island of the Galápagos archipelago the sampled individuals come from. This is a general problem with *C. nigra*: previous genetic studies have shown that the current location of a turtle is not a reliable indicator of its true origin because of human-made translocations [21,31]. For this reason, these studies concluded that a genetic assignment of lineage of origin is the most trustworthy tool to use. Our sample, which covers a wide range of *C. nigra* mitochondrial and microsatellite lineages, is appropriate with respect to this criterion. Importantly, our analysis revealed no departure from panmixy in *C. nigra*. This is the most favorable situation for studying population genomics and molecular evolution, in which any random sample of individuals is expected to provide an unbiased estimate of the species genetic diversity, irrespective of geography. We conclude that this potential concern regarding sampling is unlikely to affect our conclusions, even though they would be worth confirming based on a larger population sample.

### Divergence times and substitution rates within the chelonoidis genus

Our phylogenetic analysis revealed a significant heterogeneity in *d*_N_/*d*_S_ ratio across branches, with *C. nigra* showing the highest *d*_N_/*d*_S_ ratio of all the analyzed turtle lineages. This is consistent with the hypothesis of a reduced population size in the giant Galápagos tortoise. To what extent the high *d*_N_/*d*_S_ ratio in *C. nigra* is explained by its insularity is unclear, however, and dependent on the timing of divergence within the *Chelonoidis* genus and colonization of the Galápagos archipelago. The literature is contradictory regarding the divergence date between *C. nigra* and *C. carbonaria*. In a phylogenetic analysis of five genes in 32 Testudinoidea species and one fossil calibration, Le and McCord [28] estimated that the *C. nigra*/*C. carbonaria* split occurred 29 +/- 7 million years ago. However, from an analysis of mitochondrial DNA within the *Chelonoidis* genus and a biogeographic calibration, it was suggested that *C. nigra* diverged from its closest relative *C. chilensis* as early as 3.2 million years ago [19]. Propagating this estimate to the *C. nigra*/*C. carbonaria* pair based on their data yields an estimated age of approximately 5 million years ago for this split, that is, six times younger than the Le and McCord figure.

Our analysis of 248 nuclear genes indicates that the average amount of synonymous divergence between *C. nigra* and *C. carbonaria* is 0.052 substitutions per synonymous site (Additional file 2). Assuming a divergence as recent as approximately 5 million years for this split would imply a synonymous substitution rate of approximately 5.10^-3^ substitution per site per million years in *Chelonoidis*. This is well outside the range of nuclear synonymous substitution rate recently estimated across 132 turtle species (three nuclear genes) [27], and 20 times as high as the median rate in this study. Such a dramatic increase in substitution rate would be expected to substantially lengthen *Chelonoidis* branches in molecular phylogenies - an effect that was not conspicuous in references [26] and [28]. In contrast, the 29 million years estimate of Le and McCord, which was used in this study, would imply a plausible synonymous rate estimate of 9.10^-4^ substitution per site per million years in the *Chelonoidis* genus. One reason for the unexpectedly recent divergence times inferred by Poulakakis et al. [19] is their assumption that the *C. nigra*/*C. chilensis* split occurred at the time of the emergence of the first Galápagos Islands, that is, 2.2 to 4 million years ago. This does not need to be necessarily true: the split might have predated the emergence of the archipelago, assuming that the ancestral species that invaded the islands some Mya has gone extinct in the continent since then [18]. According to this scenario, only about 10% of the *C. nigra* branch in the tree of Figure 1 would be associated with insularity, which perhaps explains the modest *d*_N_/*d*_S_ increase in this lineage compared to, for example, the *C. carbonaria* branch.

### A typical low-N_e_ species

The average synonymous diversity in *C. nigra* was very low (π_S_ = 0.0013 to 0.0019), much lower than in the similarly analyzed European pond turtle *E. orbicularis*. Among vertebrates, levels of synonymous diversity below our *C. nigra* estimate have only been reported in the common marmoset *Callithrix jacchus* (π_S_ = 0.0012), and *Homo sapiens* (π_S_ = 0.0012) [6]. Noticeably, this top-three list of low-diversity champions includes very diverse species in terms of current abundance - the geographically restricted Galápagos tortoise, the relatively abundant, locally invasive common marmoset, and the highly successful humans.

Besides its low genome-average level of genetic polymorphism, we found an extremely high ratio of non-synonymous to synonymous diversity in *C. nigra*. Our π_N_/π_S_ estimate (0.28 to 0.32) is the highest value reported so far from genome-wide data, suggesting that purifying selection against mildly deleterious non-synonymous variants is less effective in *C. nigra* than in most living species. As compared to published values, our estimate might be slightly inflated by the absence of a reference genome in *C. nigra*, and the *de novo* prediction of ORF we performed. However, we note that a similar analysis performed in *E. orbicularis* did not yield such an elevated π_N_/π_S_ ratio. Divergence analysis consistently revealed a high *d*_N_/*d*_S_ ratio in *C. nigra*., similar to the one reported in human (Figure 1, Table 3). The analysis of flanking regions also revealed a reduced efficiency of purifying selection as compared to *E. orbicularis*.

Therefore, for all the population genomic indicators we considered, *C. nigra* showed the expected characteristics of a low-population sized species, that is, a reduced amount of genetic diversity and a weakened efficiency of selective effects. This is consistent with the idea that *C. nigra* has experienced a reduced long-term *N*_e_ as a consequence of its isolation in a restricted geographic area [14]. This result, if confirmed from other case studies, would make island endemic species a promising model for the analysis of the *N*_e_ effect on genome evolution. Besides the variable we examined in this study, one may think to investigate, for example, the complexity of the transcriptome, proteome and interactome in island *vs*. mainland species, to test the suggestion that these features primarily evolve through the long-term accumulation of neutral or slightly deleterious elements and interactions [9].

From a conservation point of view, the report of a small long-term *N*_e_ and of elevated π_N_/π_S_ and *d*_N_/*d*_S_ ratio suggests that the giant Galápagos tortoise suffers from a particularly heavy load of deleterious mutations, both fixed and polymorphic, as compared to the other turtles of this study. This might be a matter of worry. However, our own species, *H. sapiens*, is similarly affected by a substantial load of deleterious mutations [32], which have apparently not hampered its ecological success. Obviously, the giant Galápagos tortoise deserves to be protected irrespective of its level of genetic diversity and mutation load.

Our data did not reveal any departure from panmixy in *C. nigra*. No nuclear population structure between the two sampled mitochondrial lineages was found, and no significant excess of individual homozygosity was detected. This result is in apparent contradiction with the report of significant amounts of genetic differentiation between numerous pairs of *C. nigra* populations, based on a much larger sample of tortoises and 10 microsatellite loci [33]. The two studies are very different in terms of locus sampling, population sampling, and data type, and more data and analyses would be required to identify the reasons for this discrepancy. We note that the *E. orbicularis* analysis revealed a substantial excess of homozygosity and significant F_ST_ between populations, suggesting that our approach has some power to detect population differentiation when effective. At any rate, the lack of genetic differentiation genome-wide that we report between entities that recently reached species level [34] calls for a re-examination of the taxonomy in this group.

### Functional population transcriptomics

The McDonald-Kreitman approach did not reveal any evidence for adaptive amino-acid substitution in the *C. nigra* lineage. The π_N_/π_S_ ratio was higher than the *d*_N_/*d*_S_ ratio, and this was still true after low-frequency variants were accounted for. This negative result, however, might be explained by a recent drop of *N*_e_ in *C. nigra*, which would have inflated the π_N_/π_S_ ratio to a value well above its average level during the divergence between *C. carbonaria* and *C. nigra*. A global analysis of gene expression level did not reveal any specific pattern in the *C. nigra* lineage, in which the global rate of gene expression evolution did not appear to be accelerated, as compared to other turtle species. We note that these results about gene expression levels should be taken with caution because the physiological state of the sampled individuals was not properly controlled, and probably very different between species - for instance, *E. orbicularis* individuals were caught from the wild, whereas *C. nigra* individuals were sampled in zoos.

The GO-term specific π_N_/π_S_ analysis we conducted identified four terms for which the π_N_/π_S_ ratio is significantly higher in *C. nigra* than in *E. orbicularis* relative to the genomic average, that is, evidence for relaxed selective pressure in the Galápagos tortoise (Figure 5, Additional file 3: Table S1). These terms correspond to basic metabolic functions of the cell, including ‘translation’ and ‘RNA metabolic process’. We note that RNA translation is amongst the most energy-consuming cellular functions. The increased amount of functional constraint on these genes in *E. orbicularis* might be related to the necessity of substantial energetic savings in this temperate species, which enters into a deep hypometabolic state during wintering [35,36].

Two GO-slim terms, on the other hand, yielded evidence for reduced relative π_N_/π_S_ ratio in *C. nigra*, namely ‘immune system process’ and ‘response to stress’. ‘Immune system process’ is the only GO-slim term for which the *C. nigra* average (0.22) is below the *E. orbicularis* one (0.36). A high non-synonymous to synonymous ratio in immunity genes is typical and interpreted as reflecting the effect of balancing selection, that is, a selective force favoring the diversity of pathogen-responding alleles. Observing a relatively low π_N_/π value for immunity genes in *C. nigra* is therefore suggestive of a reduced pathogenic/parasitic diversity in this insular species. This is in agreement with the hypothesis of a shift from antibody-mediated, acquired immunity to cell-mediated, innate immunity in insular species [37].

‘Response to stress’ is the other GO-slim term for which a significant reduction in π_N_/π_S_ ratio was detected in *C. nigra*, most likely reflecting an increased selective constraint on these genes in the giant Galápagos tortoise. Seven of the 31*C. nigra* coding sequences associated to this category have homologues that encode for heat-shock proteins (for example, *dnaJ*), that is, chaperone proteins involved in the response to various kinds of environmental stress, such as temperature, infection, and starvation, among others (Additional file 4: Table S2). This might be related to the highly fluctuating climate of the Galápagos Islands, and the long, recurrent periods of aridity that animals must face [38]. Besides heat-shock proteins, a majority of the *C. nigra* coding sequences associated to the ‘response to stress’ term have homologues related to the control of oxidative stress (for example, peroxydase), DNA replication/repair (for example, photolyase), and apoptosis (for example, *bcl*2-associated transcription factor, Additional file 4: Table S2), that is, functions that have been linked to the regulation of ageing and longevity [39]. Our results suggest that the giant Galápagos tortoises, which can live well above 100 years in a warm, mutagenic environment, are undergoing a particularly strong selective pressure with respect to the management of oxidative molecular species and DNA damage.

## Conclusions

Our analyses of transcriptomic diversity in *C. nigra* revealed that molecular evolution in the giant Galápagos tortoise is strongly influenced by an increased rate of genetic drift, which weakens the efficiency of natural selection and creates a substantial mutation load. The relaxation of purifying selection affects most genes, regulatory regions, and functions, with the exception of genes involved in response to stress, on which selective pressure has been reinforced, presumably in response to the new environmental conditions and elongated life span in this species. This study points to insular species as a promising model to explore further the effect of genetic drift on genomic patterns, and its relationship with gene function and adaptation.

## Material and methods

### Sampling and sequencing

Blood from five adult *C. nigra*, one *C. carbonaria* and one *M. leprosa* individuals were sampled from public European zoological collections (Table 1). The sampling and animal handling have been done by veterinarians and staff of the Montpellier Zoo, Zurich Zoo, Rotterdam Zoo, and A Cupulatta Zoo according to the Code of Practice and Code of Ethics established by the European Association of Zoos and Aquarias. RNA was extracted and cDNA libraries were sequenced using Genome Analyzer II (Illumina®, see Additional file 2). Sequence reads were deposited in the NCBI Sequence Read Archive database as bioproject PRJNA230239, biosample accession SRS509366-SRS509372 (see Table 1).

### Transcriptome assembly and genotype calling

Contigs (predicted cDNAs) were assembled following reference [40]. Illumina reads were mapped to the contigs using BWA [41]. In *C. nigra*, single-nucleotide polymorphisms (SNPs) and genotypes were called using the method described in reference [42]. Dubious SNPs potentially resulting from hidden paralogy were cleaned using the method introduced in reference [22]. This method calculates the likelihood under a two-locus model (that is, assuming that two distinct genes have contributed reads which were erroneously assembled in a single contig), and discard SNPs that reveal a significant increase in likelihood, as compared to the one-locus model. Two stringency levels were tried in this analysis: stringent (likelihood ratio test *P* value <0.01) and very stringent (likelihood ratio test *P* value <0.05).

### Polymorphism analysis

For each ORF, the following statistics were calculated: per-site synonymous (π_S_) and non-synonymous (π_N_) diversity in *C. nigra*, per-individual heterozygosity, overall heterozygote deficiency (F_IT_), number of synonymous (*p*_S_) and non-synonymous (*p*_N_) segregating sites in *C. nigra*, number of synonymous (*d*_S_) and non-synonymous (*d*_S_) fixed differences between *C. nigra* and *C. carbonaria*, neutrality index NI = (*p*_N_/*p*_S_)/(*d*_N_/*d*_S_), neutrality index calculated after removing SNPs for which the minor allele frequency was below 0.2 (NI_0.2_), estimated fraction of adaptive amino-acid substitutions α = 1-NI, and its corrected version α_0.2_ = 1-NI_0.2_. An additional estimate of α, called α_EWK_, was calculated according to a method based on the inferred site frequency spectrum [29], that is, the distribution of minor allele frequency across SNPs. A similar analysis was conducted in the European pond turtle *E. orbicularis* based on a sample of 10 individuals, using the pond slider *T. scripta* as outgroup, reproducing a previously published analysis [22]. In both *C. nigra* and *E. orbicularis*, we extracted 5′ and 3′ UTR sequences from contigs in which a start and/or a stop codon had been recovered. UTR sequences of length 50 bp or more available in at least four (*C. nigra*) or eight (*E. orbicularis*) individuals were selected. UTR sequence polymorphism was estimated using the nucleotide diversity index, separately averaged across 5′ UTR and 3′ UTR sequences.

### Gene ontology analysis

The generic GO-slim ontology [23] was used to explore the distribution of the π_N_/π_S_ ratio across functional categories of genes in *C. nigra* vs. *E. orbicularis*, in search for gene sets showing a specific increase/decrease of selective pressure in one of the two species. GO-slim is a contracted version of the Gene Ontology database including a relatively small number of high-level, little-overlapping terms. A term-averaged log-transformed π_N_/π_S_ ratio was calculated for each species and each term, and compared between species. A resampling procedure was designed to quantify, for each term, the significance of the normalized difference in π_N_/π_S_ ratio between the two species (see Additional file 2).

## Competing interests

The author declare that they have no competing interests.

## Authors’ contributions

EL contributed to conceive the project and performed the population genomic analyses. YC contributed to conceive the project and generated the data. AB and VC contributed analytical tools and helped with data analysis. JR performed the phylogenomic analyses. BN contributed to conceive the project and performed the flanking sequence analyses. JML conceived and performed the gene ontology analysis. NG conceived the project and wrote the manuscript. All authors read and approved the final manuscript.

## Supplementary Material

Additional file 1: Figure S1 Mitochondrial DNA genealogy in *C. nigra*. The tree was built based on a 705-long fragment of the control region from 89*C. nigra* individuals. Sequence accession numbers and island of origin of each turtle are provided. The three major clades identified by Caccone et al. [18] are indicated. The five individuals analyzed in this study appear in red. Click here for file

Additional file 2 Details about sampling, sequencing, assembly, SNP calling, gene expression, gene ontology, and phylogenomic analyses. Click here for file

Additional file 3: Table S1 Contrasting GO-slim term-specific selective pressure between *C. nigra* and *E. orbicularis*. list of GO-slim ‘Biological process’ terms, with the number of associated contigs, their average π_N_/π_S_ ratio and its normalized version (z-score) in *C. nigra vs. E. orbicularis*. Click here for file

Additional file 4: Table S2 *C. nigra* and *E. orbicularis* coding sequences associated to GO-slim terms ‘immune process’ and ‘response to stress’. list of predicted contigs associated to the ‘immune process’ and ‘response to stress’ terms in either *C. nigra* or *E. orbicularis*, their functional annotation, length, coverage, π_N_, and π_S_. Click here for file
