# Supplementary figures and images for: Population genomics of the endangered giant Galápagos tortoise
Source: Genome Biol. 2013 Dec 16;14(12):R136. doi: 10.1186/gb-2013-14-12-r136 (PMC4053747; doi:10.1186/gb-2013-14-12-r136)

0.01

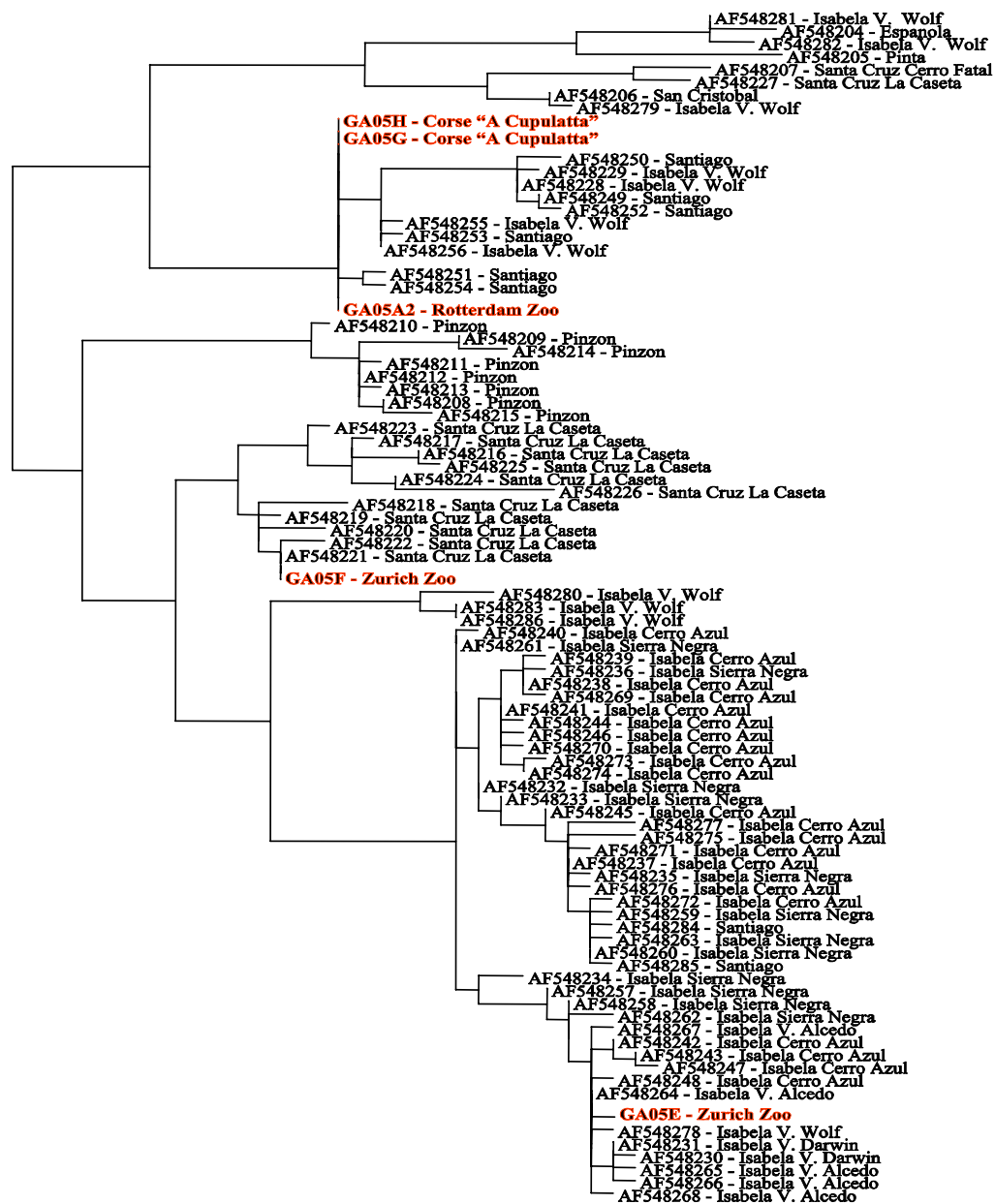

Clade d

Clade c

Clade e

Supplement: Additional file 1: Figure S1 — Mitochondrial DNA genealogy in C. nigra. The tree was built based on a 705-long fragment of the control region from 89C. nigra individuals. Sequence accession numbers and island of origin of each turtle are provided. The three major clades identified by Caccone et al. [18] are indicated. The five individuals analyzed in this study appear in red. [file gb-2013-14-12-r136-S1.pdf]
