# Supplementary material for: Population genomics of the endangered giant Galápagos tortoise
Source: Genome Biol. 2013 Dec 16;14(12):R136. doi: 10.1186/gb-2013-14-12-r136 (PMC4053747; doi:10.1186/gb-2013-14-12-r136)
Supplement: Additional file 2 — Details about sampling, sequencing, assembly, SNP calling, gene expression, gene ontology, and phylogenomic analyses. [file gb-2013-14-12-r136-S2.doc]

**Supplementary Methods**

*Sampling and sequencing*

Five adult *C. nigra* individuals (nomenclature following Turtle Taxonomy Working Group 2011 [S1]) were sampled from public European zoological collections. These individuals have been caught in the Galápagos Islands before the 1960's (predating any captive-breeding program in Europe), and kept in captivity since then. The exact sampling localities in the wild are unknown. Blood samples were taken and preserved in TriReagent BD ®, and total RNA was extracted following the protocol described in reference [S2]. For each individual, three to five µg of total RNA were used to build 3'-primed, non-normalized cDNA libraries, sequenced using Genome Analyzer II (Illumina®). A hundred bp single-end reads were produced. Low quality bases, adaptors and primers were removed using the SeqClean program (http://compbio.dfci.harvard.edu/tgi/). In *C. nigra*, a published 454 data set [42] was re-used to help with transcriptome assembly.

Four of the five *C. nigra* individuals have previously been typed for mtDNA (control region) and microsatellites [S3]. To further confirm the lineage assignment of all the sampled tortoises, including the individual sampled at the Rotterdam Zoo that has not been typed so far, control region sequences were extracted from the transcriptomic data and compared to published *C. nigra* data. Three individuals of our sample belong to mitochondrial clade *c* in reference [18] – lineage *becki*. In two of them for which microsatellite loci have been analysed, assignation to this population was confirmed [S3]. The other two individuals belong to clade *d* in reference [18] – lineages *porteri* and *vandenburghi* – and this was again confirmed by microsatellites [S3]. So the two most common mtDNA clades of *C. nigra* are represented in our sample (Figure S1).

*Transcriptome assembly*

Illumina and 454 reads were assembled into predicted cDNA contigs using a combination of the Abyss [S4] and Cap3 [S5] programs. In *C. nigra*, a published 454 data set was re-used to help with transcriptome assembly. Method D in reference [36] was followed. In this approach, 454 and Illumina reads are separately assembled then merged in a mixed assembly thanks to an additional Cap3 run. In *C. carbonaria* and *M. leprosa*, method B from reference [36] was followed (Illumina only). Open reading frames (ORF) were predicted by applying the program transcripts_to_best_scoring_ORFs.pl (courtesy of Brian Haas). Partial ORFs, in which either the start codon or the stop codon or both are missing from the sequenced fragment, were allowed. 5' and 3' untranslated regions (UTR) were respectively defined as the total contig length upstream and downstream of the predicted start and stop codon, if any.

*Genotype calling*

Illumina reads were mapped to the contigs using BWA. Potential PCR duplicates (i.e., identical reads from the same individual) were removed. Contig coverage was calculated as the summed length of reads mapping to that contig divided by contig length. Contigs less covered than an average 2.5 X per individual were discarded. SNPs and genotypes were called using the home-made program reads2snps [38]. This method estimates the sequencing error rate from the data in the maximum likelihood framework and calculates the posterior probability of genotypes assuming Hardy-Weinberg equilibrium. Genotypes of posterior probability above 0.95 were validated, provided that at least 10 reads were available for the considered position and individual. Otherwise, the data was considered as missing. SNPs identified as due to potential hidden paralogs – i.e., duplicate genes wrongly assembled into a single contig – were also marked as missing data [39].

*Outgroup inclusion*

A similar treatment was applied to the one *C. carbonaria* and the one *M. leprosa* individuals analysed in this study. Orthology relationships between *C. nigra* and *C. carbonaria* contigs were predicted using a reciprocal best BLAST hit strategy, a hit being considered as valid when alignment length was above 130 bp, sequence similarity above 80%, and e-value below e-50. *C. carbonaria* and *C. nigra* orthologues were aligned using a profile-alignment version of MACSE [S6], a program dedicated to the alignment of coding sequences and the detection of frameshifts. Contigs were only retained when no frameshift was identified by MACSE, and when the predicted ORF in *C. nigra* was longer than 200 bp. For each alignment, codon sites for which genotype data was missing in too many *C. nigra* individuals were discarded. Three data sets were built, in which the minimal number of genotyped individuals that were required to keep a site was three, four and five, respectively. Alignments made of less than 10 codon sites after cleaning were not considered further. Population genomic statistics were calculated using the Bio++ library [S7].

*Gene expression analysis*

A collection of chicken coding sequences was downloaded from the Ensembl database and taken as a reference for gene expression analysis. For each of the five turtle species for which Illumina data were available, the predicted ORFs were compared to the chicken database using BLASTP (e-value<10^-100^). For any given chicken gene, expression level in a certain turtle species was defined as the summed coverage of contigs from this species that significantly matched this gene – or zero in absence of a significant match. Gene expression levels were re-scaled so that the average level for any species was arbitrarily set to one, and log-transformed. For every pair of turtle species, the correlation coefficient, *r*, of log-transformed normalized expression level across genes was calculated. A Neighbour-Joining tree was built using *d*=1-*r*^2^ as the distance between two species [S8]:

((Mauremys_leprosa:0.61456,(Chelonoidis_nigra:0.19157,Chelonoidis_carbonaria:0.18988):0.17438):0.00437,(Trachemys_scripta:0.21357,Emys_orbicularis:0.24771):0.08298);

*Gene ontology analysis*

For each term of the GO-slim data base, we calculated a *C. nigra*-specific π_N_/π_S_ ratio, called [π_N_/π_S_]_nig_, by taking the average π_N_/π_S_ across all *C. nigra* coding sequences to which this term was associated by homology (InterProScan analysis, http://www.ebi.ac.uk/Tools/pfa/iprscan/), the average being weighted by ORF length. The expected distribution of π_N_/π_S_ for this term was obtained by re-sampling coding sequences. One thousand pseudo-replicates were built by randomly sampling in the whole set of *C. nigra* coding sequences, controlling for sequence number and average sequence length. In practice, 20,000 candidate sets sharing the same number of coding sequences as the target set were generated, candidate sets were ranked according to their similarity to the target set in terms of average sequence length, and the top 5% were selected. The average and standard deviation of log-transformed π_N_/π_S_ across pseudo-replicates were computed and a term-specific *z*-score was defined as:

$z_{\text{nig}}=\frac{\text{log}\left[\pi_{N}/\pi_{S}\right]_{\text{nig}}-\text{mean}(\text{log}\left[\pi_{N}/\pi_{S}\right]_{\text{nig}})}{\text{sd}(\text{log}\left[\pi_{N}/\pi_{S}\right]_{\text{nig}})}$, (1)

where mean() and sd() are taken across pseudo-replicates. The *z*-score measures the relative deviation of the term-specific log-transformed π_N_/π_S_ ratio from the genomic average, taking sampling variance into account. A term-specific average π_N_/π_S_ ratio, [π_N_/π_S_]_orb_, and its *z*-score, *z*_orb_, were similarly calculated in *E. orbicularis*. Our test statistics, Δ_z_, was defined as:

Δ_z_ = z_nig_ - z_orb_ (2)

Δ_z_ measures, for a given GO-slim term, the contrast in selective pressure between the two species accounting for the overall genomic trends. Δ_z_ is expected to be high and positive when the term-specific π_N_/π_S_ ratio is substantially higher than the genomic average in *C. nigra*, and/or substantially lower than the genomic average in *E. orbicularis*. A high and negative value would indicate the opposite situation, and a value close to zero would suggest the absence of a significant contrast between the two species. A *p*-value was associated to the Δ_z_ statistics by assuming that it follows a normal distribution N(0,2) under the null hypothesis of equal relative selective pressure in the two species – N(0,2) is the expected distribution of *X*_1_- *X*_2_ , where *X*_1_ and *X*_2_ are two independent standard normal deviates. A non-parametric *p*-value for Δ_z_ was also calculated based on resampling. This was achieved by re-using the pseudo-replicates pairwise, thus inferring the expected distribution of Δ_z_ in random subsets of coding sequences. To increase the size of the data set, all the contigs carrying an ORF of length >200 bp were included in this analysis, irrespective of orthology detection in outgroup species.

A similar analysis was conducted using contig relative expression level instead of π_N_/π_S_. A term-specific expression level was calculated in *C. nigra* and *E. orbicularis* for each term of the GO-slim "biological process" ontology by summing the expression levels of all associated contigs. The corresponding *z*-score and *p*-value were calculated as explained above, in search for functional categories of genes showing a significant difference in gene expression level between the two species.

*Phylogenomic analysis*

Besides the data newly generated here, we gathered published transcriptome sequence reads in the European pond slider *Emys orbicularis* [39], the red-eared slider *Trachemys scripta* [39,S9], the Hilaire's toadhead turtle *Phrynops hilari* [S10] and the loggerhead sea turtle *Caretta caretta* [S10]. In each of these species, predicted cDNAs were assembled as described above. Gene coding sequences from the fully-sequenced Chinese softshell turtle *Pelodiscus sinensis* were downloaded from Ensembl. Coding sequence alignments across the eight turtle species were obtained for the 248 genes selected in a recent phylogenomic analysis of amniotes [S10]. A reciprocal best BLAST hit strategy (program: tblastX; criteria: alignment length > 100 bp, score > 100, e-value < 1e-100, identity > 50%) was used to identify orthologues. Coding sequences were aligned using MACSE [S6]. Ambiguously aligned regions were removed manually. Alignments were concatenated before phylogenetic analysis. A phylogenetic tree was reconstructed using RaxML [S11], and between-lineages variations of the *d*_N_/*d*_S_ ratio were analyzed using the codeml program [S12], assuming either a constant or a free-to-vary *d*_N_/*d*_S_ ratio across branches. The codeml analysis performed assuming variable *d*_N_/*d*_S_ ratio across branches yielded the following branch lengths:

Non-synonymous branch lengths: (phrynops: 0.011765, (pelodiscus: 0.012814, (caretta: 0.003937, ((mauremys: 0.002681, (nigra: 0.004310, carbonaria: 0.005605): 0.003931): 0.001641, (trachemys: 0.005174, emys: 0.004152): 0.001264): 0.003253): 0.003698): 0.002078);

Synonymous branch lengths: (phrynops: 0.095428, (pelodiscus: 0.130010, (caretta: 0.039816, ((mauremys: 0.019071, (nigra: 0.021072, carbonaria: 0.032095): 0.023044): 0.014394, (trachemys: 0.028601, emys: 0.023862): 0.013173): 0.017762): 0.026244): 0.018419);

Internal nodes were assigned a divergence date based on estimates in reference [24] (*C. nigra*/*C. carbonaria* divergence) and [22] (all other nodes). Branch-specific per My synonymous substitution rates were calculated by dividing synonymous branch lengths by branch-specific divergence time.

**References**

S1. Turtle Taxonomy Working Group - van Dijk, P.P., Iverson, J.B., Shaffer, H.B., Bour, R., and Rhodin, A.G.J. (2011). Turtles of the world, 2011 update: annotated checklist of taxonomy, synonymy, distribution, and conservation status. In: Rhodin, A.G.J., Pritchard, P.C.H., van Dijk, P.P., Saumure, R.A., Buhlmann, K.A., Iverson, J.B., and Mittermeier, R.A. (Eds.). Conservation Biology of Freshwater Turtles and Tortoises: A Compilation Project of the IUCN/SSC Tortoise and Freshwater Turtle Specialist Group. Chelonian Research Monographs No. 5, doi:10.3854/crm.5.000.checklist.v4.2011

S2. Chiari Y, Galtier N (2011) RNA extraction from sauropsids blood: evaluation and improvement of methods. *Amphibia-Reptilia* 32:136-139.

S3. Russello MA et al. (2007) Lineage identification of Galápagos tortoises in captivity worldwide. *Animal Conservation* 10: 304–311.

S4. Simpson JT et al (2009) ABySS: a parallel assembler for short read sequence data. *Genome Res* 19: 1117-1123.

S5. Huang X, Madan A (1999) CAP3: A DNA sequence assembly program. *Genome Res* 9: 868-877.

S6. Ranwez V, Harispe S, Delsuc F, Douzery EJ (2011) MACSE: Multiple Alignment of Coding SEquences accounting for frameshifts and stop codons. *PLoS One* 6: e22594.

S7. Dutheil J et al. (2006) Bio++: a set of C++ libraries for sequence analysis, phylogenetics, molecular evolution and population genetics. *BMC Bioinform* 7: 188

S8. Brawand D et al. (2011) The evolution of gene expression levels in mammalian organs. *Nature* 478: 343-348.

S9. Tzika AC, Helaers R, Schramm G, Milinkovitch MC (2011) Reptilian-transcriptome v1.0, a glimpse in the brain transcriptome of five divergent Sauropsida lineages and the phylogenetic position of turtles. *Evodevo* 2: 19.

S10. Chiari Y, Cahais V, Galtier N, Delsuc F (2012) Phylogenomic analyses support the position of turtles as the sister group of birds and crocodiles (Archosauria). *BMC Biology* 10: 65.

S11. Stamatakis A, Ludwig T, Meier H (2005) RAxML-III: a fast program for maximum likelihood-based inference of large phylogenetic trees. *Bioinformatics* 21: 456-463.

S12. Yang Z (2007) PAML 4: phylogenetic analysis by maximum likelihood. *Mol Biol Evol* 24: 1586-1591.
