# Supplementary material for: Population genomics of the endangered giant Galápagos tortoise
Source: Genome Biol. 2013 Dec 16;14(12):R136. doi: 10.1186/gb-2013-14-12-r136 (PMC4053747; doi:10.1186/gb-2013-14-12-r136)
Supplement: Additional file 3: Table S1 — Contrasting GO-slim term-specific selective pressure between C. nigra and E. orbicularis. list of GO-slim ‘Biological process’ terms, with the number of associated contigs, their average πN/πS ratio and its normalized version (z-score) in C. nigra vs. E. orbicularis. [file gb-2013-14-12-r136-S3.doc]

**Table S1: Contrasting GO-slim term-specific selective pressure between *C. nigra* and *E. orbicularis*.**

|  | cat |  |  | *C. nigra* | | | *E. orbicularis* | | |  |
| --- | --- | --- | --- | --- | --- | --- | --- | --- | --- | --- |
|  |  | ID | term | # | π_N_/π_S_ | *z*-score | # | π_N_/π_S_ | *z*-score | *p*-val |
| E | 3 | GO:0006950 | response to stress | 31 | 0.110 | -2.783 | 9 | 0.048 | -0.495 | 0.046 |
| F | 3 | GO:0002376 | immune system process | 8 | 0.215 | -0.697 | 12 | 0.360 | 1.179 | 0.077 |
|  | 5 | GO:0006811 | ion transport | 51 | 0.317 | -0.761 | 36 | 0.155 | 0.515 | 0.177 |
|  | 1 | GO:0006259 | DNA metabolic process | 93 | 0.278 | -1.534 | 39 | 0.097 | -0.729 | 0.297 |
|  | 2 | GO:0044281 | small molecule metabolic process | 36 | 0.328 | -0.461 | 11 | 0.100 | 0.171 | 0.323 |
|  | 2 | GO:0009058 | biosynthetic process | 122 | 0.325 | -1.112 | 55 | 0.113 | -0.497 | 0.341 |
|  | 3 | GO:0042592 | homeostatic process | 25 | 0.570 | 0.655 | 15 | 0.274 | 1.135 | 0.362 |
|  | 4 | GO:0000902 | cell morphogenesis | 1 | 0.246 | -0.394 | 1 | 0.199 | -0.086 | 0.375 |
|  | 5 | GO:0007165 | signal transduction | 86 | 0.455 | 0.417 | 44 | 0.183 | 0.420 | 0.525 |
|  | 7 | GO:0008150 | biological_process | 149 | 0.382 | -0.262 | 77 | 0.100 | -0.389 | 0.539 |
|  | 7 | GO:0055114 | oxidation-reduction process | 87 | 0.504 | 0.784 | 42 | 0.145 | 0.700 | 0.541 |
|  | 2 | GO:0005975 | carbohydrate metabolic process | 43 | 0.405 | 0.040 | 18 | 0.061 | -0.439 | 0.653 |
|  | 4 | GO:0016043 | cellular component organization | 94 | 0.293 | -1.245 | 57 | 0.059 | -1.880 | 0.662 |
|  | 5 | GO:0022900 | electron transport chain | 9 | 0.276 | -0.543 | 6 | 0.055 | -1.135 | 0.668 |
|  | 2 | GO:0046034 | ATP metabolic process | 13 | 0.359 | -0.119 | 14 | 0.065 | -0.824 | 0.698 |
|  | 2 | GO:0006091 | generation of precursor  metabolites and energy | 13 | 0.395 | -0.028 | 4 | 0.033 | -0.917 | 0.734 |
|  | 1 | GO:0006351 | transcription, DNA-dependent | 126 | 0.311 | -1.216 | 43 | 0.050 | -2.108 | 0.735 |
|  | 7 | GO:0016310 | phosphorylation | 39 | 0.338 | -0.513 | 14 | 0.041 | -1.450 | 0.755 |
|  | 2 | GO:0051186 | cofactor metabolic process | 13 | 0.900 | 0.785 | 4 | 0.095 | -0.357 | 0.793 |
|  | 2 | GO:0045333 | cellular respiration | 9 | 0.375 | -0.158 | 9 | 0.034 | -1.390 | 0.798 |
|  | 2 | GO:0046039 | GTP metabolic process | 39 | 0.278 | -0.950 | 27 | 0.026 | -2.244 | 0.826 |
|  | 5 | GO:0006810 | transport | 160 | 0.291 | -1.723 | 71 | 0.041 | -3.110 | 0.833 |
|  | 2 | GO:0006807 | nitrogen compound metabolic  process | 28 | 0.949 | 1.483 | 15 | 0.112 | -0.103 | 0.867 |
|  | 1 | GO:0019538 | protein metabolic process | 228 | 0.363 | -0.776 | 85 | 0.055 | -2.365 | 0.877 |
| D | 1 | GO:0009117 | nucleotide metabolic process | 15 | 2.497 | 2.400 | 10 | 0.095 | -0.117 | 0.949 |
| B | 2 | GO:0009056 | catabolic process | 110 | 0.369 | -0.436 | 63 | 0.027 | -3.547 | 0.983 |
| A | 1 | GO:0006412 | translation | 129 | 0.347 | -0.756 | 133 | 0.036 | -4.891 | 0.997 |
| C | 1 | GO:0016070 | RNA metabolic process | 72 | 0.606 | 1.396 | 31 | 0.026 | -2.842 | 0.998 |

Categories:

1 Metabolism (macromolecules)

2 Metabolism (other)

3 Immunity

4 Cellular organization

5 Transport

6 Regulation

7 Generic
