# Supplementary material for: Population genomics of the endangered giant Galápagos tortoise
Source: Genome Biol. 2013 Dec 16;14(12):R136. doi: 10.1186/gb-2013-14-12-r136 (PMC4053747; doi:10.1186/gb-2013-14-12-r136)
Supplement: Additional file 4: Table S2 — C. nigra and E. orbicularis coding sequences associated to GO-slim terms ‘immune process’ and ‘response to stress’. list of predicted contigs associated to the ‘immune process’ and ‘response to stress’ terms in either C. nigra or E. orbicularis, their functional annotation, length, coverage, πN, and πS. [file gb-2013-14-12-r136-S4.doc]

**Table S4: *C. nigra* and *E. orbicularis* coding sequences associated to GO-slim terms "immune process" and "response to stress".**

| sp | term | cDNA | homologue | annotations | lg | π_N_ (%) | π_S_ (%) | cov |
| --- | --- | --- | --- | --- | --- | --- | --- | --- |
| *Cn* | str | 2093 | Heat shock protein 90/HTPG | protein folding | 2058 | 0.025 | 0.383 | 34.8 |
| *Cn* | str | 20290 | Heat shock protein 90 | protein folding | 1671 | 0.000 | 0.198 | 17 |
| *Cn* | str | 2885 | DNA repair helicase rad25 | nucleotide-excision repair; | 1356 | 0.032 | 0.000 | 11.6 |
| *Cn* | str | 2763 | Heat shock protein DnaJ | protein folding; response to heat | 1122 | 0.000 | 0.244 | 26.2 |
| *Cn* | str | 16109 | Catalase 3 | response to oxidative stress | 948 | 0.054 | 0.000 | 36 |
| *Cn* | str | 20299 | Heat shock protein 90 | protein folding | 831 | 0.091 | 0.000 | 24 |
| *Cn* | str | 28908 | Heat shock protein 90 | protein folding | 714 | 0.000 | 0.000 | 25.8 |
| *Cn* | str | 5951 | UV excision repair protein rad23 | nucleotide-excision repair | 687 | 0.000 | 0.163 | 35.2 |
| *Cn* | str | 16321 | rad50 | DNA repair | 684 | 0.000 | 0.000 | 7.6 |
| *Cn* | str | 357 | Heat shock protein 90 | protein folding | 609 | 0.000 | 0.000 | 23 |
| *Cn* | str | 21558 | Bcl-2-associated transcription factor 1 | induction of apoptosis | 579 | 0.116 | 0.000 | 5.4 |
| *Cn* | str | 3204 | rad51-interacting protein | DNA repair | 465 | 0.055 | 0.000 | 20.8 |
| *Cn* | str | 124271 | Heat shock protein 90 | protein folding | 348 | 0.000 | 0.000 | 28.6 |
| *Cn* | str | 2447 | Gluthathione peroxidase | response to oxidative stress | 333 | 0.000 | 1.015 | 38.8 |
| *Cn* | str | 18829 | Cathelicidin | defense response | 327 | 0.000 | 1.126 | 25.4 |
| *Cn* | str | 20442 | Tbf5 | nucleotide-excision repair | 282 | 0.000 | 0.000 | 27.4 |
| *Cn* | str | 28025 | Catalase | response to oxidative stress | 273 | 0.000 | 0.421 | 17.4 |
| *Cn* | str | 22890 | Testis-expressed sequence 15 protein | regulation of double-strand break repair | 237 | 0.000 | 0.392 | 9.4 |
| *Cn* | str | 7494 | Probable helicase senataxin | double-strand break repair | 231 | 0.000 | 0.000 | 7.4 |
| *Cn* | str | 21121 | Bcl-2-associated transcription factor 1 | induction of apoptosis | 183 | 0.000 | 0.000 | 7.4 |
| *Cn* | str | 13447 | rad14: DNA repair protein | nucleotide-excision repair | 174 | 0.000 | 0.000 | 7.4 |
| *Cn* | str | 18330 | DNA-3-Methyladenine glycosylase | base-excision repair | 153 | 0.000 | 0.000 | 10 |
| *Cn* | str | 26397 | DNA_photolyase | DNA repair | 135 | 0.000 | 0.000 | 15.2 |
| *Cn* | str | 239 | Checkpoint protein Rad1 | DNA damage checkpoint; DNA repair | 90 | 0.000 | 0.000 | 6.8 |
| *Cn* | str | 12780 | Checkpoint protein Rad17/Rad24 | DNA repair; cell cycle | 81 | 0.000 | 0.000 | 5.2 |
| *Cn* | str | 26326 | Transcription factor IIH subunit | DNA repair; regulation of transcription | 69 | 0.000 | 0.000 | 6.4 |
| *Cn* | str | 130335 | Bcl-2-associated transcription factor 1 | induction of apoptosis | 63 | 0.000 | 0.000 | 5 |
| *Cn* | str | 3310 | DNA repair polymerase | DNA repair | 57 | 0.000 | 0.000 | 5.6 |
| *Cn* | str | 16797 | DNA Helicase | double-strand break repair | 51 | 0.000 | 0.000 | 5.8 |
| *Cn* | str | 8013 | cytokine receptor | inflammatory response | 48 | 0.000 | 0.000 | 5 |
| *Cn* | str | 11998 | Probable helicase senataxin | double-strand break repair | 45 | 0.000 | 9.630 | 4.8 |
| *Eo* | str | 1145 | Cathelicidin | defense response | 139 | 0.002 | 0.021 | 374 |
| *Eo* | str | 15879 | UV excision repair protein rad23 | nucleotide-excision repair | 25 | 0.000 | 0.000 | 2.8 |
| *Eo* | str | 16581 | Glutathione peroxidase | response to oxidative stress | 112 | 0.001 | 0.000 | 51.2 |
| *Eo* | str | 2549 | Tbf5 | nucleotide-excision repair | 86 | 0.000 | 0.000 | 21.1 |
| *Eo* | str | 2565 | Heat shock protein DnaJ | protein folding; response to heat | 227 | 0.000 | 0.000 | 12.3 |
| *Eo* | str | 2987 | Heat shock protein DnaJ | protein folding; response to heat | 124 | 0.000 | 0.006 | 12.4 |
| *Eo* | str | 637 | catalase | response to oxidative stress | 56 | 0.000 | 0.000 | 10.2 |
| *Eo* | str | 7038 | Heat shock protein 90 | protein folding | 663 | 0.000 | 0.013 | 17.6 |
| *Eo* | str | 8786 | rad51-interacting protein | DNA repair | 61 | 0.000 | 0.000 | 14 |
| *Cn* | imm | 11353 | MHC II alpha | antigen processing and presentation | 89 | 0.007 | 0.037 | 10.8 |
| *Cn* | imm | 11486 | HLA-DR antigens-associated invariant chain CD74 | antigen processing and presentation | 225 | 0.001 | 0.001 | 57 |
| *Cn* | imm | 11798 | Tumor necrosis factor receptor | apoptotic process; immune response | 212 | 0.000 | 0.003 | 13.2 |
| *Cn* | imm | 18054 | Tumor necrosis factor | immune response | 72 | 0.000 | 0.005 | 7.6 |
| *Cn* | imm | 257 | Interleukin enhancer-binding factor 2 | immune response | 364 | 0.000 | 0.002 | 35 |
| *Cn* | imm | 28538 | Alpha-hemoglobin stabilizing protein | protein folding; protein stabilization | 29 | 0.000 | 0.024 | 9.8 |
| *Cn* | imm | 404 | HLA class II, gamma subunit | antigen processing and presentation | 132 | 0.002 | 0.002 | 60 |
| *Cn* | imm | 585 | MHC class II-related | antigen processing and presentation | 71 | 0.011 | 0.035 | 17.6 |
| *Eo* | imm | 14948 | BETA-2 Microglobulin (MHC lass I-related) | immune response | 119 | 0.000 | 0.000 | 47 |
| *Eo* | imm | 15820 | MHC class I-related | antigen processing and presentation | 73 | 0.002 | 0.000 | 8.4 |
| *Eo* | imm | 159 | MHC class I-related | antigen processing and presentation | 19 | 0.000 | 0.058 | 19.3 |
| *Eo* | imm | 16251 | MHC class I-related | antigen processing and presentation | 164 | 0.001 | 0.001 | 42.9 |
| *Eo* | imm | 18101 | MHC class I-related | antigen processing and presentation | 34 | 0.018 | 0.021 | 6.6 |
| *Eo* | imm | 19330 | MHC class I-related | antigen processing and presentation | 35 | 0.006 | 0.015 | 7 |
| *Eo* | imm | 22068 | MHC class I-related | antigen processing and presentation | 32 | 0.004 | 0.000 | 7.8 |
| *Eo* | imm | 2246 | MHC class I-related | antigen processing and presentation | 124 | 0.016 | 0.047 | 15.3 |
| *Eo* | imm | 23039 | MHC class I-related | antigen processing and presentation | 23 | 0.018 | 0.026 | 6 |
| *Eo* | imm | 820 | HLA class II, gamma chain | antigen processing and presentation | 41 | 0.000 | 0.007 | 22.4 |
| *Eo* | imm | 94985 | MHC CLASS II alpha chain | antigen processing and presentation | 22 | 0.008 | 0.021 | 3 |
| *Eo* | imm | 986 | Interleukin enhancer-binding factor 2 | immune response | 219 | 0.000 | 0.004 | 23 |

*Cn*: *Chelonoidis nigra*

*Eo*: *Emys orbicularis*

str : response to stress

imm: immunity

annotations: GO terms associated to each coding sequences (biological process)

lg: length of the cleaned coding sequence alignment

cov: per site, per individual average sequencing coverage
